# Supplementary material for: Tracheophyte genomes keep track of the deep evolution of the Caulimoviridae
Source: Sci Rep. 2018 Jan 12;8:572. doi: 10.1038/s41598-017-16399-x (PMC5766536; doi:10.1038/s41598-017-16399-x)
Supplement: Supplementary file 1 — Supplementary Figures [file 41598_2017_16399_MOESM1_ESM.pdf]

Tracheophyte genomes keep track of the deep evolution of the *Caulimoviridae*

Authors

Seydina Diop[1], Andrew D.W. Geering[2], Françoise Alfama-Depauw[1], Mikaël Loaec[1], Pierre-Yves Teycheney[3] and Florian Maumus[1*]

Supplementary figures

Supplementary figure 1

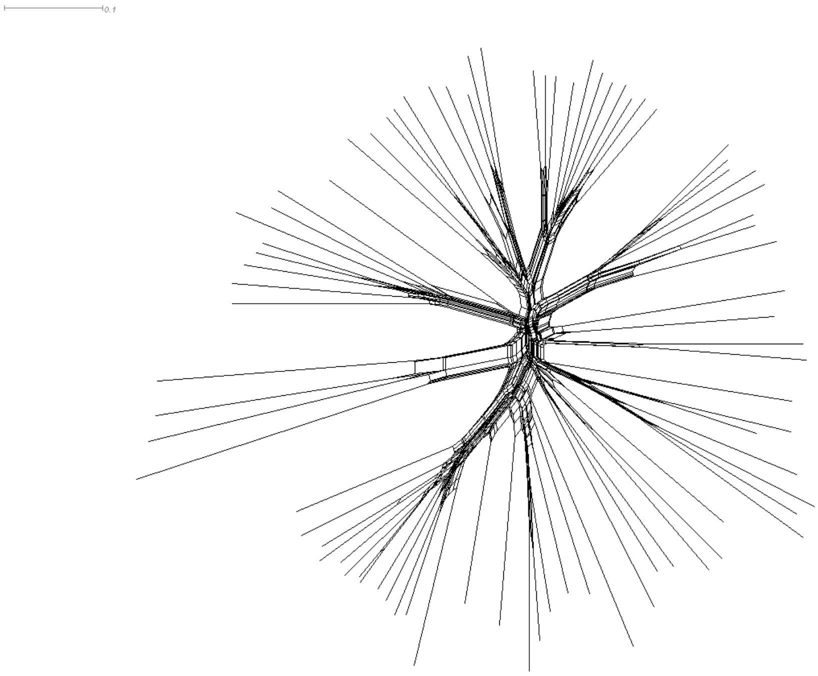

Supplementary Figure 1: Overview of the phylogenetic network used to build Figure 1.

Supplementary figure 2

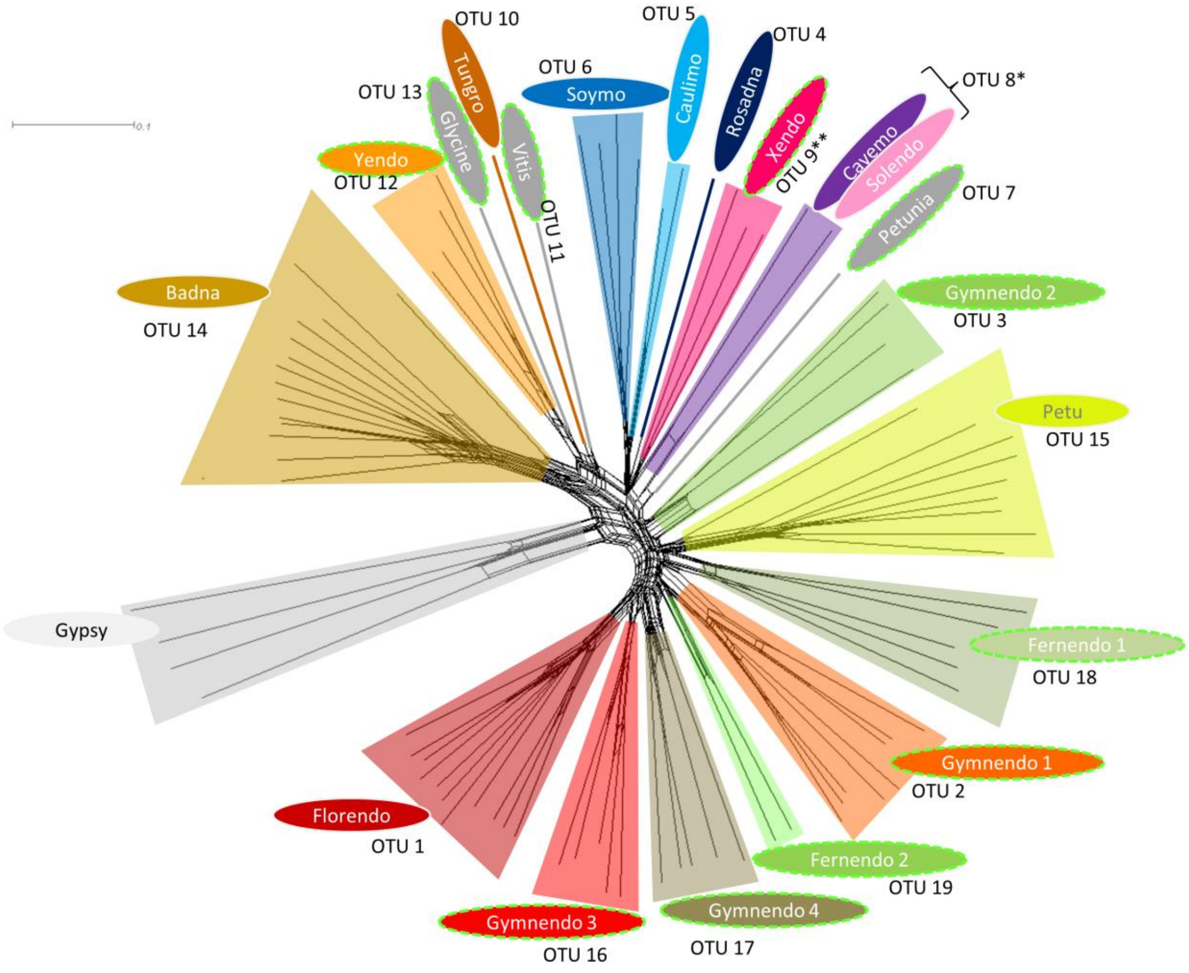

Supplementary Figure 2: ECRT ORFs collected from ferns cluster as two novel OTUs. Representative sequences identified in fern genomes were appended to the collection of sequences represented in Figure 1. The resulting library has been re-aligned with MUSCLE and phylogenetic network was built using SplitsTree. The branches containing fern sequences have been empirically grouped into two novel OTUs (OTU 18 and OTU 19).
