# Supplementary material for: Tracheophyte genomes keep track of the deep evolution of the Caulimoviridae
Source: Sci Rep. 2018 Jan 12;8:572. doi: 10.1038/s41598-017-16399-x (PMC5766536; doi:10.1038/s41598-017-16399-x)
Supplement: Supplementary file 3 — Supplementary Table 1 [file 41598_2017_16399_MOESM3_ESM.pdf]

	Division or order	Family	Species	Common name	Source	# of ERCT open reading frames (	# of ECRT loci	ECRT loci / Mb
Green algae	Volvocales	Volvocaceae	*Volvox carteri*		Phytozome v11	0	0	NA
	Chlorophyta	Chlamydomonadaceae	*Chlamydomonas reinhardtii*		Phytozome v11	0	0	NA
	Chlorophyta	Coccomyxaceae	*Coccomyxasp.C-169*		Phytozome v11	0	0	NA
	Charophyta	Characeae	*Chara braunii*		Phytozome v11	0	0	NA
Basal land plants	Lycophyta	Selaginellaceae	*Selaginella moellendorffii*		Phytozome v11	0	0	NA
	Bryophyta	Funariaceae	*Physcomitrella patens*	Spreading earthmoss	Phytozome v11	0	0	NA
Gymnosperms	Ginkgophyta	Ginkgoaceae	*Ginkgo biloba*	Gingko	http://gigadb.org/dataset/100209	187	571	0,056449648
	Pynophyta	Pinaceae	*Picea abies*	Norway spruce	http://congenie.org/	417	664	0,055248525
	Pynophyta	Pinaceae	*Picea glauca*	White spruce	http://congenie.org/	804	1396	0,055863526
	Pynophyta	Pinaceae	*Pinus taeda*	Loblolly pine	http://congenie.org/	1058	2364	0,115136272
Angiosperms	Amborellales	Amborellaceae	*Amborella trichopoda*	Amborella	Phytozome v11	422	698	1,044508136
	Alismatales	Zosteraceae	*Zostera marina*	Common eelgrass	https://bioinformatics.psb.ugent.be/gdb/zostera/annotation/v2.2/nuclear/	0	0	0
	Liliales	Dioscoreaceae	*Dioscorea alata*	Yam	https://www.ncbi.nlm.nih.gov/nuccore/CZHE00000000.2	63	94	0,15146657
	Asparagales	Orchidaceae	*Phalaenopsis equestris*	Orchid	https://www.ncbi.nlm.nih.gov/nuccore/APLD00000000.1, another one is https://www.ncbi.nlm.nih.gov/genome/11403	17	44	0,044738057
	Poales	Poaceae	*Zea mays B73*	Maize	Ensembl 33	0	0	0
	Poales	Poaceae	*Setaria italica*	Foxtail millet	Phytozome v11	9	13	0,032426306
	Poales	Poaceae	*Brachypodium distachyon*	Purple false brome	Ensembl 33	13	19	0,070167346
	Poales	Poaceae	*Oryza sativa*	Rice	Phytozome v11	36	41	0,109755527
	Poales	Poaceae	*Oryza brachyantha*		Ensembl 33	0	0	0
	Zingiberales	Musaceae	*Musa acuminata*	Banana	http://banana-genome-hub.southgreen.fr/	22	22	0,054248897
	Arecales	Arecaceae	*Elaeis guineensis*	Oil palm	https://www.ncbi.nlm.nih.gov/nuccore/ASJS00000000.1	7	39	0,036898614
	Poales	Bromeliaceae	*Ananas comosus*	Pineapple	https://genomevolution.org/CoGe/NotebookView.pl?nid=937	10	12	0,031990854
	Brassicales	Brassicaceae	*Brassica rapa*	Oilseed	Ensembl 33	7	7	0,023434902
	Brassicales	Brassicaceae	*Schrenkiella parvula*		http://thellungiella.org/data/	0	0	0
	Brassicales	Brassicaceae	*Eutrema salsugineum*		Phytozome v11	44	54	0,226439532
	Brassicales	Brassicaceae	*Arabidopsis thaliana*	Thale cress	TAIR10	0	0	0
	Brassicales	Brassicaceae	*Arabidopsis lyrata*		Phytozome v11	28	31	0,168689923
	Brassicales	Brassicaceae	*Arabis alpina*	Alpine rock-cress	https://www.ncbi.nlm.nih.gov/nuccore/JNGA00000000.1	12	16	0,056657708
	Brassicales	Brassicaceae	*Capsella rubella*	Pink shepherd's-purse	Phytozome v11	1	5	0,038439387
	Brassicales	Brassicaceae	*Tarenaya hassleriana*	Spider flower	https://www.ncbi.nlm.nih.gov/nuccore/AOUI00000000.1	8	16	0,07300928
	Brassicales	Caricaceae	*Carica papaya*	Papaya	Phytozome v11	0	0	0
	Malvales	Sterculiaceae	*Theobroma cacao*	Cacao	Phytozome v11	4	26	0,078549162
	Malvales	Malvaceae	*Gossypium raimondii*	Cotton	Phytozome v11	51	151	0,201849661
	Sapindales	Rutceae	*Citrus sinensis*	Sweet orange	Phytozome v11	332	575	2,277119658
	Myrtales	Myrtaceae	*Eucalyptus grandis*	Eucalypt	Phytozome v11	189	351	0,548040878
	Malpighiales	Salicaceae	*Populus trichocarpa*	Poplar	Phytozome v11	23	50	0,118221705
	Malpighiales	Euphorbiaceae	*Ricinus communis*	Castor bean	Phytozome v11	465	672	1,994323275
	Malpighiales	Euphorbiaceae	*Hevea brasiliensis*	Rubber tree	https://www.ncbi.nlm.nih.gov/nuccore/LVXX00000000.1	253	524	0,405079459
	Fagales	Fagaceae	*Quercus robur*	Oak	Plomion et al.	194	264	0,334066748
	Violales	Cucurbitaceae	*Cucumis sativus*	Cucumber	Phytozome v11	2	3	0,015600953
	Cucurbitales	Cucurbitaceae	*Citrullus lanatus*	Watermelon	ftp://www.icugi.org/pub/genome/watermelon/97103/	4	13	0,040453475
	Rosales	Rosaceae	*Fragaria vesca*	Strawberry	Phytozome v11	46	85	0,441156312
	Rosales	Rosaceae	*Malus x domestica*	Apple	Phytozome v11	127	187	0,214725449
	Rosales	Rosaceae	*Prunus persica*	Peach	Phytozome v11	75	95	0,422904579
	Rosales	Cannabaceae	*Cannabis sativa*	Cannabis	https://www.ncbi.nlm.nih.gov/nuccore/MNPR00000000.1	7	9	0,015362985
	Fabales	Fabaceae	*Cajanus cajan*	Pigeon pea	https://www.ncbi.nlm.nih.gov/nuccore/AGCT00000000.1	100	140	0,25072461
	Fabales	Fabaceae	*Lotus japonicus*		ftp://ftp.kazusa.or.jp/pub/lotus/lotus_r3.0/	104	101	0,256057493
	Fabales	Fabaceae	*Medicago truncatula*	Barrelclover	ftp://ftp.jcvi.org/pub/data/m_truncatula/Mt4.0/Assembly/	5	9	0,023135294
	Fabales	Fabaceae	*Glycine max*	Soybean	Phytozome v11	160	240	0,251202952
	Saxifragales	Crassulaceae	*Kalenchoe fedtschenkoi*		Phytozome v11	31	34	0,140871414
	Rhamnales	Vitaceae	*Vitis vinifera*	Grape	Phytozome v11	84	164	0,348785751
	Proteales	Nelumbonaceae	*Nelumbo nucifera*	Sacred lotus	https://www.ncbi.nlm.nih.gov/nuccore/AQOG00000000.1	1	16	0,022597262
	Solanales	Solanaceae	*Solanum pennellii*	Wild tomato	ftp://ftp.solgenomics.net/genomes/	239	398	0,454630826
	Solanales	Solanaceae	*Solanum lycopsericum*	Tomato	ftp://ftp.solgenomics.net/genomes/	154	369	0,500218863
	Solanales	Solanaceae	*Solanum tuberosum*	Potato	ftp://ftp.solgenomics.net/genomes/	164	340	0,536192857
	Solanales	Solanaceae	*Capsicum annuum - Zunla*	Bell pepper	https://www.ncbi.nlm.nih.gov/nuccore/ASJU00000000.1	967	1467	0,542243037
	Solanales	Solanaceae	*Solanum melongena*	Eggplant	ftp://ftp.solgenomics.net/genomes/	37	146	0,17524736
	Solanales	Solanaceae	*Petunia axillaris*	White moon petunia	ftp://ftp.solgenomics.net/genomes/	145	176	0,144508934
	Solanales	Solanaceae	*Petunia inflata*	Violet petunia	ftp://ftp.solgenomics.net/genomes/	149	182	0,151701508
	Solanales	Solanaceae	*Nicotiana tabacum*	Tobacco	https://www.ncbi.nlm.nih.gov/nuccore/AYMY00000000.1	414	776	0,219344693
	Solanales	Solanaceae	*Nicotiana sylvestris*	Woodland tobacco	https://www.ncbi.nlm.nih.gov/nuccore/493002186	246	421	0,205588152
	Solanales	Solanaceae	*Nicotiana tomentosiformis*		https://www.ncbi.nlm.nih.gov/nuccore/497187505	176	331	0,201517158
	Scrophulariales	Scrophulariaceae	*Mimulus guttatus*	Monkey flower	Phytozome v11	49	56	0,193178151
	Rubiales	Rubiaceae	*Coffea canephora*	Coffee	http://coffee-genome.org/coffeacanephora	113	155	0,328866128
	Asterales	Asteraceae	*Conyza canadensis*	Horseweed	https://www.ncbi.nlm.nih.gov/nuccore/JSWR00000000.1	1	5	0,015329655
	Asterales	Asteraceae	*Lactuca sativa*	Lettuce	https://www.ncbi.nlm.nih.gov/nuccore/AFSA00000000.1	4	22	0,01940622
	Theales	Actinidiaceae	*Actinidia chinensis*	Kiwi fruit	https://www.ncbi.nlm.nih.gov/nuccore/AONS00000000.1	53	91	0,150608106
	Ericales	Ericaceae	*Vaccinium corymbosum*	Blueberry	http://www.igbquickload.org/blueberry/V_corymbosum_Aug_2015/	12	13	0,045512188
	Ericales	Ericaceae	*Vaccinium macrocarpon*	Cranberry	https://www.ncbi.nlm.nih.gov/nuccore/JOTO00000000.1	4	12	0,028944927
	Caryophyllales	Amaranthaceae	*Beta vulgaris*	Sugar beet	http://bvseq.molgen.mpg.de/Genome/Download/RefBeet-1.2/	15	20	0,038621505
	Ranunculales	Ranunculaceae	*Aquilegia caerulea*	Columbine	Phytozome v10	67	73	0,249083539
	Proteales	Proteaceae	*Macadamia integrifolia*	Macadamia nut	http://www.ebi.ac.uk/ena/data/view/FLKO01000000	13	27	0,06031307
Ferns	Polypodiopsida	Gleicheniales	*Dipteris conjugata*		http://digitalcommons.usu.edu/fern_genome/2/	11	50	no complete genome
	Polypodiopsida	Cyatheales	*Plagiogyria formosana*		http://digitalcommons.usu.edu/fern_genome/2/	3	10	no complete genome
	Polypodiopsida	Pteridaceae	*Ceratopteris richardii*		http://digitalcommons.usu.edu/fern_genome/2/	0	20	no complete genome
	Polypodiopsida	Dennstaedtiaceae	*Pteridium aquilinum*	Eagle fern	http://digitalcommons.usu.edu/fern_genome/2/	1	36	no complete genome
	Polypodiopsida	Eupolypods II	*Cystopteris protrusa*	Lowland bladderfern	http://digitalcommons.usu.edu/fern_genome/2/	5	12	no complete genome
	Polypodiopsida	Eupolypods I	*Polypodium glycyrrhiza*	Licorice fern	http://digitalcommons.usu.edu/fern_genome/2/	1	6	no complete genome
